# The Rising Threat of Mucormycosis: Oman’s Experience Before and During the COVID-19 Pandemic

**DOI:** 10.3390/jof10110796

**Published:** 2024-11-15

**Authors:** Amina Al-Jardani, Adil Al-Wahaibi, Azza Al Rashdi, Bram Spruijtenburg, Noora AlBulushi, R. Sandhya Rani, Hanan AlKindi, Fatma Al-Yaquobi, Bader Al-Rawahi, Asma AlBalushi, Saleh Al Azri, Jacques F. Meis, Iman AlBuloshi, Seif Al-Abri, Ahmed Al-Harrasi, Abdullah M. S. Al-Hatmi, Amal Al Maani

**Affiliations:** 1 Central Public Health Laboratories, Center for Disease Control and Prevention, Ministry of Health, Muscat 100, Oman; arashdi24@gmail.com (A.A.R.); nooradi-b@hotmail.com (N.A.); sasu_mct@yahoo.co.in (R.S.R.); drhananalkindi@gmail.com (H.A.);salehabdullahalazri@gmail.com (S.A.A.); 2 Surveillance Department, Center for Disease Control and Prevention, Ministry of Health, Muscat 100, Oman; adilwahaibi@gmail.com; 3 Radboudumc-CWZ Center of Expertise for Mycology, 6532 SZ Nijmegen, The Netherlands; b.sprujtenburg@cwz.nl (B.S.); jacques.meis@gmail.com (J.F.M.); a.alhatmi@unizwa.edu.om (A.M.S.A.-H.); 4 Department of Medical Microbiology and Immunology, Canisius-Wilhelmina Hospital (CWZ)/Dicoon, 6532 SZ Nijmegen, The Netherlands; 5 Communicable Disease, Center for Disease Control and Prevention, Ministry of Health, Muscat 100, Oman; fatmayaquobi@gmail.com (F.A.-Y.); baderalrawahi4@gmail.com (B.A.-R.); 6 Infectious Diseases Unit, Internal Medicine Department, Sultan Qaboos University Hospital, Muscat 123, Oman; thebrightsoul2@gmail.com; 7 Institute of Translational Research, Cologne Excellence Cluster on Cellular Stress Responses in Aging-Associated Diseases (CECAD) and Excellence Center for Medical Mycology, University of Cologne, 50923 Cologne, Germany; 8 Surveillance Department, Disease Surveillance and Control, Directorate General of Health Services South Batinah Governorate, Ministry of Health, Muscat 100, Oman; emanalbeloushi9@gmail.com; 9 Infectious Diseases Unit, Department of Medicine, Royal Hospital, Muscat 111, Oman; salabri@gmail.com; 10 Natural and Medical Sciences Research Center, University of Nizwa, Nizwa 616, Oman; alharrasi@unizwa.edu.om; 11 Center for Disease Control and Prevention, Ministry of Health, Muscat 100, Oman; amalsaifalmaani@gmail.com

**Keywords:** mucormycosis, epidemiology, COVID-19, Oman

## Abstract

Mucormycosis is a rare, severe fungal infection mainly affecting immunocompromised individuals. Because of limited data on its epidemiology in Oman, we present this national, multicentric, retrospective review that includes all cases of proven mucormycosis between 2006 and 2022 in Oman. There were 51 cases of mucormycosis reported in Oman. The annual incidence of mucormycosis was 0.38–0.69 cases per million population before COVID-19. During the pandemic, the incidence rose significantly to 1.76 in 2020, 5.31 in 2021, then decreased to 0.87 per million population in 2022. Diabetes was observed in 82.4% (*n* = 42) of the cases, COVID-19 in 47.1% (*n* = 24), and other chronic diseases in 72.6%. The use of steroids was reported in 33.3% (*n* = 17) and many patients (64.7%, *n* = 33) had multiple risk factors. The overall mortality rate was 41.2% (*n* = 21) and most deaths occurred within a month of diagnosis. Mortality rate among patients diagnosed with COVID-19 was 58.3% (14/24). Survival analysis showed a statistically significant association between COVID-19 status and patient survival (*p* = 0.024). Annual incidence of mucormycosis in Oman rose during the pandemic. This study highlights the epidemiological features of mucormycosis and emphasizes the importance of its inclusion in the national notifiable communicable diseases priority list as well as the importance of enhancing diagnostic capacities to detect and improve patient outcomes.

## 1. Introduction

Mucormycosis is a fungal infection caused by fungal species belonging to the order Mucorales. They are ubiquitous organisms, found in soil, plants, and decaying matter. The infection is acquired through inhalation or ingestion of spores or by traumatic inoculation of skin and soft tissue. The infection hallmark is characterized by angioinvasions, which lead to infarction and thrombosis of affected tissues [1,2].

The most common Mucorales causing this angioinvasive infection belong to the genera *Rhizopus* spp., *Mucor* spp., and *Lichtheimia* spp. (formerly *Absidia* spp. and *Mycocladus* spp.). Other genera of Mucorales, such as *Rhizomucor*, *Saksenaea*, *Cunninghamella*, and *Apophysomyces*, are less common. *Rhizopus arrhizus* (previously *R. oryzae*) is the most common species, responsible for nearly 60% of mucormycosis cases in humans and accounting for 90% of the rhino-orbital-cerebral (ROC) form [3].

A recent systematic review by Jeong et al. showed that the most common clinical presentation is rhino-orbital-cerebral mucormycosis (ROCM) and this was observed in 34% of the cases, followed by cutaneous (22%) and pulmonary mucormycosis (20%) [2]. Disseminated disease was reported in 13% of the patients. In addition, for gastrointestinal mucormycosis, infection was mostly confined to the stomach, intestine, and/or colon (74%), with liver involvement in 22% of the patients [2].

Regarding risk factors, uncontrolled diabetes mellitus (DM) is considered the most common risk factor for this infection globally [1,2]; however, hematologic malignancies and organ transplant are the leading risk factors in Europe and the USA [1]. Mucorales fungi are the second-most-common filamentous fungal pathogen after *Aspergillus*, leading to invasive fungal disease in patients with malignancies and transplantation [4]. Other risk factors include corticosteroid use with a few case reports of mucormycosis resulting from even a short course (5–14 days) of steroid therapy, especially in people with DM. Interestingly, a prospective study by the European Confederation of Medical Mycology (ECMM) reported that 46% of patients received corticosteroids within the month before diagnosis [5].

The exact incidence of this rare disease is unknown as there are few published population-based studies of mucormycosis [1]. Its prevalence in the general population was previously reported as 0.005 to 1.7 per million population [2,6]. However, the incidence of mucormycosis in India was reported to be 0.14/1000 in diabetic patients, which is 80 times higher than the reported incidence in other parts of the world [6]. This may be due to the high prevalence of DM in India. In one study, DM was the underlying disease in 54–76% of mucormycosis cases, with 8–22% presenting with diabetic ketoacidosis [7]. The prevalence of mucormycosis in Oman is unknown as it is not yet a notifiable infection.

With the unprecedented COVID-19 pandemic, the rate of mucormycosis surged. COVID-19-associated mucormycosis (CAM) is increasingly reported from several countries, most commonly from India, possibly due to the high rate of DM [8]. Several other case reports and case series were reported from other countries as well [9,10,11].

There are specific pathophysiological features of COVID-19 that may predispose an individual to secondary fungal infections. First, there is immune dysregulation with reduced numbers of T lymphocytes, CD4 + T cells, CD8 + T cells, and markedly higher levels of interleukin (IL)-2 receptor, IL-6, IL-10, and tumor necrosis factor-alpha. Second, there is a propensity of SARS-CoV-2 to cause extensive pulmonary disease and subsequent alveolo-interstitial pathology may enhance the risk of invasive fungal infections, specifically those with a primary pulmonary entry such as mucormycosis [12].

Given the high mortality of mucormycosis as well as the significant mortality of COVID-19, co-infections have raised the alarm globally and become a public health threat especially in low- and middle-income countries [13].

As there is limited data on the epidemiology of mucormycosis in Oman, our study aims to describe the demographics, clinical presentation, and outcomes of patients with invasive mucormycosis as well as characterize the common causative agents and identify the risk factors predisposing the development of mucormycosis in the pre- and post-COVID-19 era.

## 2. Materials and Methods

### 2.1. Sample and Data Collection

We conducted a national retrospective case review including all cases of invasive mucormycosis between 2006 and 2022 to assess the epidemiology, associated risk factors with outcomes, and to compare the rate of infection in the pre- and post-COVID-19 periods.

The patient demographic data, clinical presentation, and presence of chronic disease—including comorbidities such as hypertension, ESRD, coronary artery disease, dyslipidemia, asthma, and others—were collected. Additionally, data on underlying risk factors—including diabetes, steroid use, immunosuppression, trauma, and other predisposing medical conditions—as well as the outcomes were collected. These data were extracted using the Electronic Health Information System (Alshifa) and the Laboratory Information System at the Central Public Health Laboratories.

Only proven cases of mucormycosis were included and these were defined based on the European Organization for Research and Treatment of Cancer/Mycoses Study Group (EORTC/MSG) definitions for invasive fungal infections [14].

### 2.2. Statistical Analysis

Descriptive statistics were employed to analyze categorical variables, which were tabulated using percentages. Age was categorized into three groups: less than 40 years old, 40 to 60 years old, and more than 60 years old. The presence of risk factors was summarized for all patients, with the total number of risk factors listed. The length of hospital stay was treated as a continuous variable and analyzed using the mean and standard deviation (SD).

A trend analysis of case occurrences over the past 16 years was conducted to identify any significant patterns or changes in case frequency over time.

The Kaplan–Meier survival curve was constructed to evaluate the survival probabilities of patients, stratified by various factors including the presence or absence of DM, age, COVID-19 status, and the accumulation of multiple risk factors. This method allows for the assessment of the impact of these variables on patient survival over the study period.

### 2.3. Laboratory Investigations

Mucormycosis was identified by histopathological examination and/or microbiological examination.

The specimens were inoculated on Sabouraud agar (Oxoid, Hampshire, UK) plates and incubated for two weeks at room temperature and 37 °C. Identification of the Identification of Mucorales isolates was carried out by macroscopic examination—including the rate of growth, colony appearance, and reverse color—and by microscopic examination of the growth using lactophenol blue. In addition, MALDI-TOFF (Maldi Biotyper MBT Compass 4.1.100, Bruker Daltonics GmbH, Bremen, Germany) was used for identification [15]. The macroscopic and microscopic morphological features of the isolates were studied following the standard procedures such as slide culture on potato dextrose agar and assessment of growth at 30, 37, 40, and 45 °C.

### 2.4. Molecular Identification

All isolates with a MALDI-TOF identification score of ≤2 were subjected to molecular identification to ensure accuracy and assess the antifungal susceptibility given the rising number of cases during the COVID-19 pandemic.

Prior to DNA extraction, isolates were cultured on Sabouraud dextrose agar (Oxoid, Hampshire, UK) at 35 °C for two days. DNA extraction and purification were conducted with the MagNA Pure 96 instrument and accompanying reagents as described earlier [16]. The internal transcribed spacer (ITS) was amplified from the extracted and purified DNA with ITS-1 and ITS-4 primers [17]. Amplicons were purified with the D-Pure protocol (Nimagen, Nijmegen, The Netherlands) and Sanger sequencing was performed with a 3500 XL genetic analyzer (Applied Biosystems, Foster City, CA, USA). Resulting ITS sequences were compared to reference strains submitted to the National Center for Biotechnology Information (NCBI) nucleotide database. The following sequences were used as controls: *R. arrhizus* (NR_103595.1), *R. arrhizus* var. *delemar* (JN943002.1), *Rhizopus microsporus* var. *chinensis* (NR_149337.1), *Rhizopus stolonifer* var. stolonifer (AB113023.1), *Mucor circinelloides* (NR_126116.1), *Mucor racemosus* (JN205991.1), *Cunninghamella bertholletiae* (JN205879.1), *Cunninghamella elegans* (JN205889.1), *Saksenaea erythrospora* (KM102733.1), *Saksenaea vasiformis* (JN206282.1), *Apophysomyces elegans* (JN206279.1), *Apophysomyces variabilis* (NR_130683.1), *Lichtheimia corymbifera* (MH863088.1), *Lichtheimia ramosa* (MH858566.1), and *Basidiobolus heterosporus* (MH858801.1). Sequence alignment and phylogenetic tree visualization were conducted as previously described [16]. All ITS sequences generated during the current study were deposited at the NCBI Genbank database under accession numbers PP788902–PP788910.

### 2.5. Antifungal Susceptibility Testing (AFST)

Using broth microdilution, the minimum inhibitory concentrations (MICs) were determined in accordance with the Clinical and Laboratory Standards Institute document M38-A2 [18]. Amphotericin B (Sigma-Aldrich, St. Louis, MO, USA), itraconazole (Janssen Pharmaceutica, Tilburg, The Netherlands), posaconazole (Merck, Darmstadt, Germany), isavuconazole (Basilea pharmaceutica, Basel, Switzerland), micafungin (Astellas, Ibaraki, Japan), and anidulafungin (Pfizer) were tested against *Mucorales* strains. Final concentrations of antifungal agents in the wells ranged from 0.016 to 16 µg/mL for amphotericin B, itraconazole, posaconazole, and isavuconazole, and 0.008 to 8 µg/mL for anidulafungin and micafungin.

Ethical approval: this study was approved by the Central Research Ethical Committee at the Ministry of Health, Oman, IRA Number (MoH/CSR/21/25129).

## 3. Results

**Figure 1:**
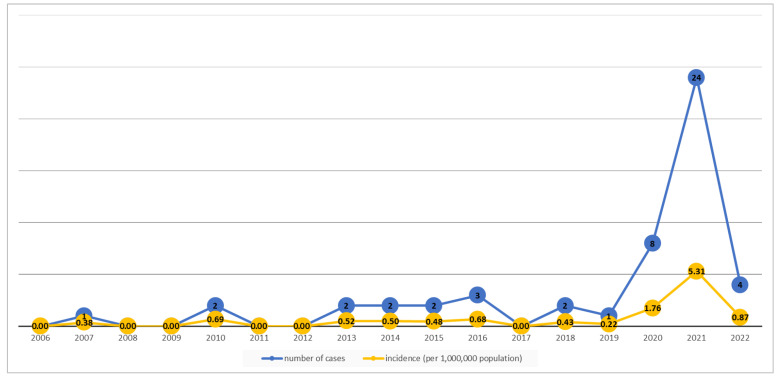
Number of cases and incidence (per 1,000,000 population) of confirmed mucormycosis cases in 2006–2022.

A total of 51 cases of mucormycosis were observed in Oman between 2006 and 2022. The annual incidence of mucormycosis was 0.38–0.69 cases per million population in the period prior to the COVID-19 pandemic. During the pandemic, the incidence rose significantly to 1.76, with a sharp increase to 5.31 in 2021 and then a decrease to 0.87 per million in 2022 (Figure 1).

Most cases were male (70.6%, *n* = 36). The distribution of age groups was as follows: patients younger than 40 years constituted 27.5% (*n* = 14) of the cases, patients who were 40 to 60 years old accounted for 41.8% (*n* = 21), and those older than 60 years represented 31.4% (*n* = 16) (Table 1).

The cases were predominantly from the North Batinah region (35.3%, *n* = 18), followed by Muscat (23.5%, *n* = 12), which are the most populated regions of the country. Other regions with reported cases included Ad Dahirah (9.8%, *n* = 5), both South and North Batinah (each 9.8%, *n* = 5), South Sharqiyah (7.8%, *n* = 4), Dhofar (5.9%, *n* = 3), Ad Dakhiliyah (3.9%, *n* = 2), Al Buraymi (2.0%, *n* = 1), and South Sharqiyah (2%, *n* = 1) (Supplementary Materials, Figure S1).

Nearly all cases (98.0%, *n* = 50) were inpatients (IPD). Regarding nationality, Omani nationals constituted most cases (82.4%, *n* = 42), followed by Indians (9.8%, *n* = 5), Bangladeshis (3.9%, *n* = 2), Pakistanis (2%, *n* = 1), and Sri Lankans (2%, *n* = 1).

A high prevalence of DM was observed among the cases (82.4%, *n* = 42). Nearly half of the patients had COVID-19 (47.1%, *n* = 24), and a significant number had other chronic diseases (72.6%, *n* = 37). The use of steroids was reported in 33.3% (*n* = 17) of the cases, and other forms of immunosuppression were present in 3.9% (*n* = 2). A vast majority of the patients (64.7%, *n* = 33) had multiple risk factors. Among the COVID-19-affected cases, 23/24 (95.8%) were diabetic, 10/24 received steroids, and 5 had other risk factors and or chronic comorbidity in addition to DM (Table 1).

Surgical management was employed in 77.6% (*n* = 38) of the cases, while systemic antifungal treatment with liposomal amphotericin B (L-AMB) was almost universally administered (98%, *n* = 50).

Over 70% (36/51) were culture positive, while 33.3% (17/51) were diagnosed based on the histopathological findings and clinical and radiological evidence of infection. The most common site for infection was ROC and was diagnosed in 68.2% (35/51) of the cases followed by sinuses and cutaneous in 13.7%. Gastrointestinal and pulmonary mucormycosis was diagnosed in 7.8% of the cases. Among the COVID-19-associated mucormycosis cases, 87.5% (21/24) had ROC mucormycosis infections. The most common species isolated from invasive mucormycosis was *Rhizopus* spp., accounting for 66.7% (24/36) of the total culture-positive cases and 82.4% of the *causative* spp. among culture-positive COVID-19 cases (Table 2).

Regarding patient outcomes, the overall mortality rate was 41.2% (*n* = 21/51) and most deaths occurred within the first month of infection. Mortality among patients diagnosed with COVID-19 was 58.3% (14/24) and among the non-COVID-19 cases was 25.9% (7/27).

The mean length of stay for patients was 30.7 days, with a SD of 59.7 days (Table 1).

**Figure 2:**
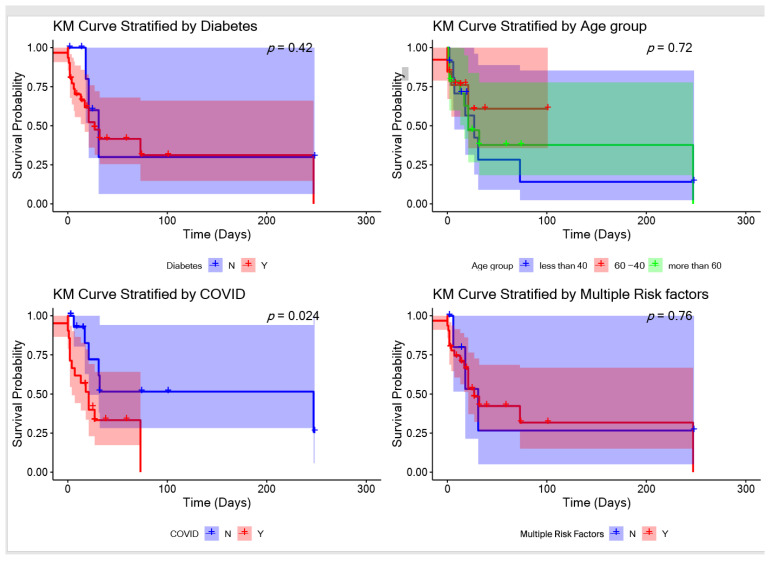
Kaplan–Meier survival curves of all patients stratified by the presence or absence of diabetes, age, COVID-19 (*p* = 0.024), and multiple risk factors.

The Kaplan–Meier survival curve was used to determine the influence of various factors on patient survival. The analysis revealed a statistically significant association between COVID-19 status and patient survival (*p* = 0.024), indicating that COVID-19 was a significant factor in survival outcomes. Other factors such as age, DM, and the presence of multiple risk factors did not show a statistically significant impact on survival (Figure 2).

Among the patients who survived, 60% (18/30) had documented complications including loss of vision, orbital exenteration, neurological deficits, osteomyelitis, and trigeminal neuralgia.

**Figure 3:**
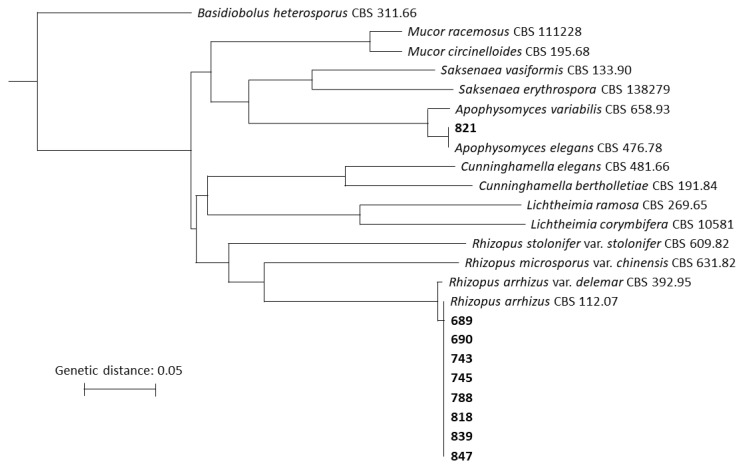
Phylogenetic tree based on multiple sequence alignment of ITS sequences. Isolates indicated in bold originate from the current study. The tree was rooted to *Basidiobolus heterosporus*.

Molecular species identification was performed on nine isolates and was based on ITS sequencing. By aligning generated sequences to several common Mucorales species, eight isolates (689, 690, 743, 745, 788, 818, 839, and 847) were identified as *R. arrhizus* and one isolate (821) as *Apophysomyces elegans* (Figure 3). There was 100% correlation between the phenotypic and MALDI-TOF identification.

In vitro AFST according to the CLSI guidelines was performed on eight *R. arrhizus* isolates against six antifungals. Resulting MICs demonstrated the highest in vitro activity against amphotericin B and was the lowest for the echinocandins (Table 3).

## 4. Discussion

This is the first and largest national, multicenter study describing the epidemiology of invasive mucormycosis in the Sultanate of Oman and highlights the impact of COVID-19 on the incidence and outcomes of this severe fungal infection. Our data show that the disease was rare before the COVID-19 pandemic, where only two to three cases were identified yearly, and with an annual incidence of mucormycosis of 0.38–0.69 cases per million population; however, during the pandemic, the incidence rose significantly to 1.76 per million in 2020, followed by a sharp increase observed in 2021 to 5.31 per million. The incidence subsequently decreased to 0.87 per million in 2022 after introduction of the COVID-19 vaccination.

The geographical distribution of cases in Oman was the highest two governates, with most cases being found in North Batinah followed by Muscat, most likely because these two regions are the most populated.

Globally, the incidence of mucormycosis is significantly variable across different regions of the world and is underestimated as it is not a notifiable disease. The prevalence is rising, mainly driven by the increase in cases in low- and middle-income countries. For example, India has one of the highest reported prevalence rates of mucormycosis, estimated to be around 0.14 cases per 1000 population, which is approximately 80 times higher than that in developed countries [8]. Similarly, there are few studies from the Middle East and the Gulf Cooperation Council region about the incidence and epidemiology of mucormycosis comparing the pre- and post-COVID eras. Most information comes from case reports, small case series, and retrospective reports [19]. The limited surveillance systems focusing on fungal infections and the lack of diagnostic capacities might have contributed to underreporting. A study from Kuwait estimated over 23 cases annually, and 22 (95.7%) of these with no underlying diseases apart from DM [20]. A retrospective multicenter study including all patients with clinical and pathological evidence of mucormycosis in Saudi Arabia from January 2009 to December 2019 showed that the mean age was 42 years. The most common site of infection was cutaneous (27%), followed by isolated sinusitis (21%), and then pulmonary and ROCM (each 18%). The most common isolated species were *Rhizopus* (50%) and *Mucor* (15%). However, no data on the actual incidence, time trend, or risk factors were highlighted [21].

Regarding the different species causing mucormycosis, several studies showed that *Rhizopus* was the most frequently isolated genus followed by *Lichtheimia* [22,23,24]. Apart from the study by Guinea et al. showing that *Cunninghamella* spp. is the most common species in Spain [25], the other studies regarding species distribution showed similar findings.

In the global review by Jeong et al., *Rhizopus* spp., *Lichtheimia* spp., and *Mucor* spp. accounted for 75% of all cases [2]. In a prospective European study, *Rhizopus* spp. were isolated in 34% of cases, *Lichtheimia* spp. in 19%, and *Mucor* spp. in 19% [5]. Similarly, in the RetroZygo study from France, *Rhizopus* spp. were the causative agents in 52%, while the second most common genus was *Lichtheimia* (29%) [26].

In India, *R. arrhizus* is the most common species [8] followed by *Apophysomyces* spp. [27,28]. New species are emerging, including *Rhizopus homothallicus* [29], *Thamnostylum lucknowense* [30], *Mucor irregularis* [31], and *Saksenaea erythrospora* [32]. These findings were similar to our study with the *Rhizopus* species as the most isolated pathogen followed by *Mucor* species.

Most of the mucormycosis reports were from India, especially in COVID-19 patients who were most commonly reported to have rhino-orbital/rhino-cerebral mucormycosis. Those patients were diabetic and had corticosteroid therapy for controlling the severity of COVID-19, leading to increased fatality in such cases and complicating the pandemic scenario [33].

Of the total cases of mucormycosis reported here, 42 cases (82.4%) had DM, of which 5 cases were diagnosed with new-onset diabetes, aligning with many worldwide studies [7,16,19,21,22,23]. A systematic review of mucormycosis cases published between January 2000 and January 2017 identified DM as a well-recognized comorbidity associated with 40% of cases [34]. Several studies conducted in India found that DM was a risk factor in 54–76% of mucormycosis cases [8]. A study from Saudi Arabia showed that people with diabetes and hematologic malignancy accounted for 48% and 42.4% of the patient population, respectively [19]. A study from Kuwait showed 95.7% (*n* = 22) of cases of mucormycosis were partly associated with diabetes, had no other underlying diseases, and only 4.3% (*n* = 1) were immunocompromised by cancer [16]. A recent 14-year study from a tertiary care center in Lebanon found that 67.4% of the patients had hematologic malignancies while 34.9% had DM [35]. Additionally, a study conducted in 13 European countries involving 230 cases of mucormycosis reported that hematologic malignancies were the most common underlying diseases (44%), while DM was the sole predisposing factor in 9% of the cases [5]. This implies that hematologic malignancies are an important underlying cause in high-income countries. Our cohort study identified only two immunosuppressed patients: one with myelodysplastic syndrome on chemotherapy and the other one being a post-liver transplant patient on immunosuppressants. In total, 64.7% of our mucormycosis patients had multiple risk factors. Common chronic diseases reported in our study were hypertension and end-stage renal disease.

Our study showed that there was an upsurge of cases of mucormycosis after the declaration of the COVID-19 pandemic with a total of 36 cases, of which 24/36 (66.6%) were CAM. A systematic review studied 101 cases of mucormycosis in COVID-19 patients, of which 82% were from India, 80% were diabetic, and 76.3% were treated with corticosteroids [10]. Among our cohort of CAM cases, 23 had diabetes and/or other risk factors. Patients with diabetes are at increased risk of severe COVID-19 due to low phagocytic activity, endothelial dysfunction, and increased hypercoagulation in addition to acidosis and hyperglycemia, which consecutively lead to an increased risk of fungal infections [36,37].

COVID-19 infection is associated with impaired iron metabolism and hyperferritinemia along with hyperglycemia and acidosis, which further hinders phagocytosis, suppresses the activity of polymorphonuclear (PMN), and enhances the susceptibility to mucormycosis, severe infection, and higher mortality [12,37,38]. Also, steroid use in COVID-19 patients compromises the immune system further. Steroid use is identified in 33.3% (*n* = 17) of the cases, of which 10 cases were receiving steroids for COVID-19 management.

The mortality rate varied among studies conducted in distinct populations showing how different factors have different impacts on survival. While Koffi et al. and Prakash et al. found a mortality rate of 38% [8,39], Rothe et al. [40] found that the crude mortality rate reached 100% among critically ill patients with mucormycosis. On the other hand, a study by Camara-Lemarroy et al. in Mexico showed a mortality rate of 50% in patients with rhino-cerebral mucormycosis who received polyene antifungal therapy, and survivors were significantly younger, less likely to have diabetes, and had higher levels of serum albumin on admission than non-survivors [41]. Among our population, patients with ROC had the highest mortality (*n* = 16), followed by pulmonary and GI infection, with two cases each, and one case involving skin and soft tissue infection.

Shamithra et al. showed that the outcome varies according to the antifungal drugs given: out of 3749 patients who received amphotericin B monotherapy, the mortality rate was 31.5%; among those who received amphotericin B and azoles (843), mortality rate was 6.6%; and the mortality rate was 17.2% among the 250 patients who received posaconazole [42]. In our study, the combination was administered to critically ill patients and those who are unable to tolerate higher doses of L-AMB due to side effects such as renal failure. Among those patients who received the combination antifungal therapy, 64% (*n* = 7/11) improved while 36% (*n* = 4/11) did not survive; however, the sample size is too small to conclude if this is statistically significant.

In another study by Jestin et al. in 26 patients with mucormycosis, investigations showed that 31% required surgical intervention, 81% required invasive mechanical ventilation, 69% required vasopressors, and 35% renal replacement therapy. The same study also found that the intensive care units and hospital mortality rates were 77% and 88%, respectively [43].

A systematic review by Shen et al. showed that mortality rates from mucormycosis in hematology patients was 61% and was significantly lower in high-income countries and among patients who received a combination of medical and surgical treatment as well as those with isolated disease compared with the disseminated form [44].

A study in post-transplant patients with mucormycosis showed 90-day and 1-year mortality rates of 36.3% and 63.4%, respectively, with disseminated disease having the highest rates at both time points. Other factors with negative impact on the 90-day mortality were treatments with >3 immunosuppressive drugs and the presence of diabetes [45].

In our study, the mortality rate was 41.8%, lower than many studies, and this might be explained by the fact that most of the patients (75%) underwent additional surgical management that positively affected survival rates. The mortality was 36.5% (*n* = 14/38) among the patients who received surgical and antifungal therapy. Other data of higher survival rates include a broad study reviewing 929 reported cases with a survival rate of 57% with surgery, 61% with only amphotericin B, and 70% for amphotericin B plus surgery; in addition, a study by Roden et al. found the overall survival rate was higher (70.2%) with surgical debridement plus posaconazole and amphotericin B than with antifungal therapy alone (32.4%) in a cohort of 174 renal transplant recipients with mucormycosis [46]. Indeed, a combination of surgery and antifungal therapy is the treatment of choice for invasive mucormycosis [47]. In our study, no other factors apart from COVID-19 were found to have an impact on the survival outcome.

The mortality rate of mucormycosis in COVID-19 cases has been described in many studies. In a systemic review including 30 case reports/case series pooling data retrieved from 99 patients with CAM, most cases were reported from India (72%) and showed a mortality rate of 34% [48].

Muthu et al. showed a mortality rate of 32.2% among COVID-19 patients in India [49], while the all-cause-mortality rate was reported as 50% from a study in the USA [38]. Similar to the latter study, a study from Egypt during the COVID-19 pandemic showed a mortality rate of 49% [50], similar to our mortality rate of 59% in comparison to 25.9% in non-COVID-19 patients.

Our study has some limitations, including its retrospective nature, the relatively small number of cases due to low prevalence of the disease, non-notification of cases, and low clinical suspicion. In addition, this study only included cases that were managed at Ministry of Health hospitals and did not include cases from university or military hospitals. The other limitation is the possibility of underdiagnosis. To diagnose mucormycosis is challenging and relies on the identification of the culprit organism in tissue by histopathology and fungal culture [47]. We found 70.6% of our cases to be culture positive, while 29.4% (13/51) were diagnosed based on histopathology, clinical, and radiological evidence of infection. However, the invasive nature of tissue sampling for testing and the limited tissue quantities make the diagnosis difficult; this is in addition to tissue handling, sampling, and prior use of antifungal therapy, which might lead to negative culture results in up to 50% of cases [51]. Similarly, obtaining a diagnosis on histopathological grounds is challenging and rarely there is a misidentification of Mucorales as *Aspergillus* spp. [47].

Nevertheless, this was the largest national study conducted in Oman to date to highlight the epidemiological features of this life-threatening infection. The results emphasize the importance of including this infection in the national notifiable diseases list and stress improving diagnostic capacities to detect and better characterize the culprit agent, which will further improve patient outcomes.

## Figures and Tables

**Table 1:** Characteristics of mucormycosis cases (*n* = 51), Oman, 2006–2022.

Category	Variable	Number (*n* = 51)	Percentage
Sex	Male	36	70.6
Age group	<40	14	27.5
	40–60 years	21	41.8
	>60	16	31.4
Region	Ad Dakhiliyah	2	3.9
	Ad Dahirah	5	9.8
	Al Buraymi	1	2.0
	Dhofar	3	5.9
	Muscat	12	23.5
	N. Batinah	18	35.3
	N. Sharqia	4	7.8
	S. Batinah	5	9.8
	S. Sharqiyah	1	2.0
Country of origin	Bangladesh	2	3.9
	India	5	9.8
	Oman	42	82.4
	Pakistan	1	2.0
	Sri Lanka	1	2.0
Diabetes		42	82.4
COVID-19 infection		24	47.1
Other chronic diseases		37	72.6
Steroid use		17	33.3
Other immunosuppression		2	3.9
Multiple risk factors		33	64.7
Surgical management		38	77.6
Systemic antifungal		50	98.0
Death (overall)		21	41.2
Death among COVID-19 patients		14	58.3
Length of stay (in days): mean (SD)	30.7 (59.7)		

**Table 2:** Pathogens causing mucormycosis and the type of infection (*n* = 51), Oman, 2006–2022.

	Genus	No. of Cases (*n* = 51)	%
Pathogens causing mucormycosis	*Absidia* spp.	2	3.9
	*Apophysomyces* *elegans*	1	1.9
	*Cunninghamella* spp.	2	3.9
	*Lichtheimia* spp.	1	1.9
	*Mixed fungal* spp.	1	1.9
	*Mucor indicus*	1	1.9
	*Mucor* spp.	4	7.
	*Rhizopus microsporus*	1	1.9
	*Rhizopus arrhizus*	17	33.3
	*Rhizopus* spp.	6	11.7
Type and site of infection	Gastrointestinal	4	7.8
	Others	7	13.7
	Pulmonary	4	7.8
	ROC	35	68.6
	Soft tissue	1	1.9

**Table 3:** In vitro MICS for eight *Rhizopus arrhizus isolates*. MICS in µg/mL.

ID	AMB	ITC	POS	ISA	AFG	MFG
689	0.125	0.5	0.5	2	≥8	≥8
690	0.063	2	1	1	≥8	≥8
743	0.063	2	0.5	0.5	≥8	≥8
745	0.063	1	0.5	2	≥8	≥8
788	0.125	2	0.25	1	≥8	≥8
818	0.063	0.5	1	1	≥8	≥8
839	0.063	1	0.5	2	≥8	≥8
847	0.063	1	0.25	1	≥8	≥8

Abbreviations: AMB, amphotericin B; ITC, itraconazole; POS, posaconazole; ISA, isavuconazole; AFG, anidulafungin; MFG, micafungin.

## Data Availability

Data are contained within the article.

## Supplementary Materials

The following supporting information can be downloaded at: https://www.mdpi.com/article/10.3390/jof10110796/s1. Figure S1: distribution of mucormycosis cases per governorates.
