# Supplementary figures and images for: The Rising Threat of Mucormycosis: Oman’s Experience Before and During the COVID-19 Pandemic
Source: J Fungi (Basel). 2024 Nov 15;10(11):796. doi: 10.3390/jof10110796 (PMC11595873; doi:10.3390/jof10110796)

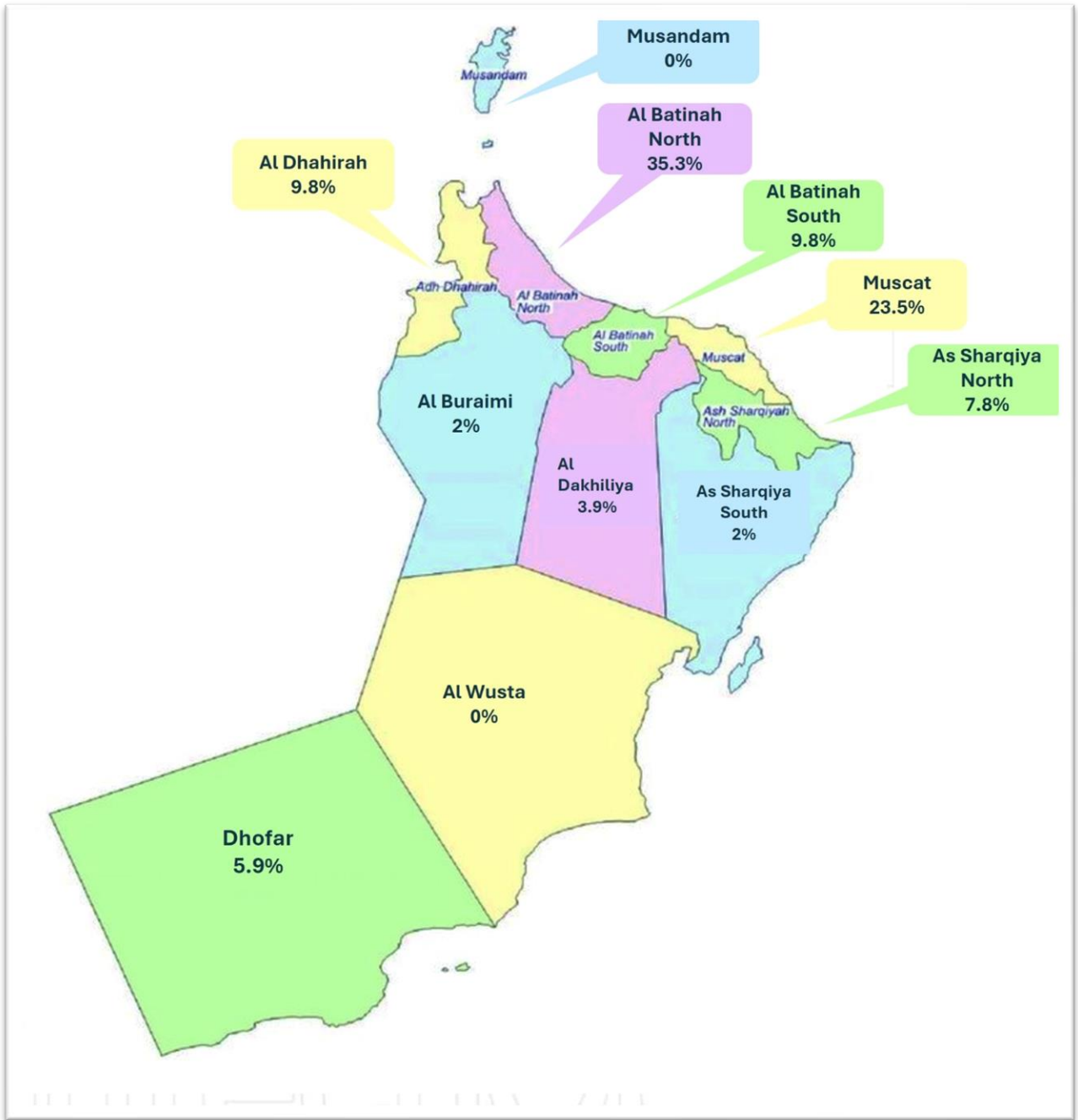

**Supplementary Figure 1:** distribution of mucormycosis cases per governorates.

Supplement: Supplementary file 1 [file jof-10-00796-s001.zip › jof-3273052-supplementary.pdf]
